# Medication adherence in obsessive-compulsive disorders and evaluation of effective adherence support strategies

**DOI:** 10.3389/fpsyt.2025.1642622

**Published:** 2025-09-12

**Authors:** Sophia Pöppel, Sina Ziegler, Klara Bednasch, Elisabeth Kohls, Christine Rummel-Kluge

**Affiliations:** ^1^ Department of Psychiatry and Psychotherapy, Faculty of Medicine, University of Leipzig, Leipzig, Germany; ^2^ Clinic for Child and Adolescent Psychiatry, DRK Clinics Berlin Westend, Berlin, Germany; ^3^ Department of Psychiatry and Psychotherapy, University Hospital Leipzig, Leipzig, Germany

**Keywords:** psychiatric patients, obsessive-compulsive disorder (OCD), medication adherence, therapeutic drug monitoring (TDM), selective serotonin reuptake inhibitors (SSRIs)

## Abstract

**Introduction:**

Medication adherence is a critical component in the treatment of psychiatric conditions such as obsessive-compulsive disorder (OCD). Poor adherence is associated with an increased risk of relapse, subsequent (re)hospitalization, and prolonged remission, which ultimately leads to a worse prognosis. This study aimed to assess medication adherence over time in individuals with OCD, identify predictors, and gather patient-reported strategies to maintain adherence.

**Methods:**

This study surveyed *N* = 100 patients recruited in the outpatient department of a university medical center in Leipzig, Germany, between January 2019 and January 2020 (Ethics Committee approval number: 332/18-ek; date of approval: 25 September 2018). Medication adherence was assessed using indirect (i.e., Drug Attitude Inventory, Medication Adherence Rating Scale) and direct methods (i.e., therapeutic drug monitoring via serum drug concentration). Additionally, the participants reported strategies they found helpful for maintaining adherence.

**Results:**

The participants exhibited mild impairments in various aspects of functioning despite relatively functional daily lives. Most were prescribed selective serotonin reuptake inhibitors (SSRIs), with a subset receiving combination therapy for treatment-resistant cases. Medication adherence was classified into three categories: 24.4% of the participants were good adherers, 62.2% were partially adherent, and 13.4% were non-adherent. Therapeutic drug monitoring (TDM) indicated that 84.6% of the participants had drug levels within the therapeutic range. Concerns about side effects and doubts regarding the efficacy of the medication were commonly reported, which might contribute to suboptimal adherence. However, no significant associations were found between adherence and sociodemographic or clinical variables, which suggested the need for a more comprehensive approach considering psychosocial factors. Behavioral strategies for maintaining adherence (e.g., incorporating medication into daily routines) were preferred and rated as helpful, while invasive monitoring methods were largely rejected.

**Discussion:**

This study highlights the importance of a multifaceted approach to improving medication adherence in individuals with OCD. While SSRIs remain the primary pharmacological treatment, a significant portion of patients still struggle with adherence. Although TDM provides valuable insights into drug levels, it may not fully capture adherence behavior due to metabolic and behavioral variability. Addressing patient concerns about side effects and medication efficacy, alongside implementing behavioral strategies that integrate medication into daily routines, may improve adherence and enhance treatment outcomes.

## Introduction

The World Health Organization defines adherence as “the extent to which a person’s behavior – e.g., taking medication, following a diet, and/or executing lifestyle changes – corresponds with agreed recommendations from a health care provider” (1). Adherence to medication includes initiation (e.g., the first intake of the drug as prescribed), implementation (e.g., the extent of medication intake compared to medically prescribed dosage), and discontinuation (e.g., ceasing medication for any reasons (2);. Nonadherent behavior can manifest in several ways. For example, patients may decide not to take their medication at all, take it at different times than prescribed, or take incorrect dosages (e.g., under-dosing and over-dosing). They may also interrupt therapy indefinitely or discontinue treatment altogether (3). Several risks are associated with poor medication adherence, including relapses and potential rehospitalization, as well as delayed remission with a reduced quality of life (1). Consequently, nonadherence poses a significant barrier to effective medical treatment in the 21^st^ century. Chronic diseases, in particular, require long-term medication adherence to improve prognosis and quality of life. However, various studies have indicated that the average adherence rate among patients with chronic illnesses (e.g., hypertension and diabetes mellitus) is approximately 50% (1, 4–6). Hence, approximately half of those with adequate access to healthcare do not follow medical instructions to optimize their health. Moreover, the rate of nonadherence among patients with chronic psychiatric conditions is even higher (7–9), with 74% of this patient group discontinuing prescribed medication within 18 months for various reasons (e.g., side effects and perceived inefficacy (10);). Despite these general trends, relatively little research has examined medication adherence in individuals with obsessive-compulsive disorder (OCD). According to the ICD-10 (11), this psychiatric condition is characterized by two primary criteria: recurring intrusive ideas, thoughts, or impulses (e.g., obsessions) and repetitive actions (e.g., compulsions), which are both associated with a high level of distress or dysfunction. Most affected individuals recognize the senselessness of their symptoms due to persistent insight during the course of the illness. The associated shame may delay initial contact with the psychiatric system and, consequently, the initiation of effective therapy (12). A previous study demonstrated that the average duration between symptom onset and diagnosis is 12.78 years. Following diagnosis, an additional 1.45 years typically pass before the initiation of treatment (13).

Guideline-recommended treatment approaches for OCD include both cognitive-behavioral therapy (CBT) with exposure and response prevention (ERP) and pharmacotherapy with selective serotonin reuptake inhibitors (SSRIs) as first-line treatments, which can be used individually or in combination depending on patient preference, symptom severity, or accessibility (14). Participants receiving antipsychotic augmentation were classified as treatment-resistant based on clinical judgment and their pharmacological regimen, although no formal staging criteria were applied. In line with previous findings (15, 16), supporting the efficacy of augmentation with risperidone and aripiprazole – and to a lesser extent quetiapine – the addition of one of these agents to an SSRI was considered a valid augmentation strategy. Few studies have systematically evaluated medication adherence in OCD, particularly with regard to influencing factors, individual support strategies, and patient resources. This study aimed to evaluate medication adherence in patients with OCD through indirect (questionnaire-based) and direct (TDM-based) methods, assess attitudes toward pharmacotherapy, and explore individual strategies for supporting adherence.

## Materials and methods

### Participants and procedure

Recruitment occurred at the psychiatric outpatient department, Department of Psychiatry and Psychotherapy at the University of Leipzig, between January 2019 and February 2020, with two survey time points within one year (i.e., initial survey T0 and follow-up investigation T1). Patients currently receiving outpatient treatment for OCD were invited to participate in the study. Inclusion criteria were being over 18 years of age, a diagnosis of OCD according to the ICD-10, diagnosis code F42 (11), the ability to provide informed consent, the ability to complete the questionnaire independently, sufficient German language skills, and adequate vision and literacy. The study was approved by the Ethical Committee of the Medical Faculty of Leipzig University (Ethics Committee approval number: 332/18-ek; date of approval: 25 September 2018) and was conducted as a web-based survey. Further details about the recruitment and participants can be found elsewhere (13).

### Recruitment

A total of 113 patients were invited to participate in the study. Of these, *N* = 100 patients (88.5%) provided informed consent and contributed data for inclusion in the subsequent analysis. Where relevant, missing data and variations in participant numbers were reported accordingly.

### Measures

**Figure 1:**
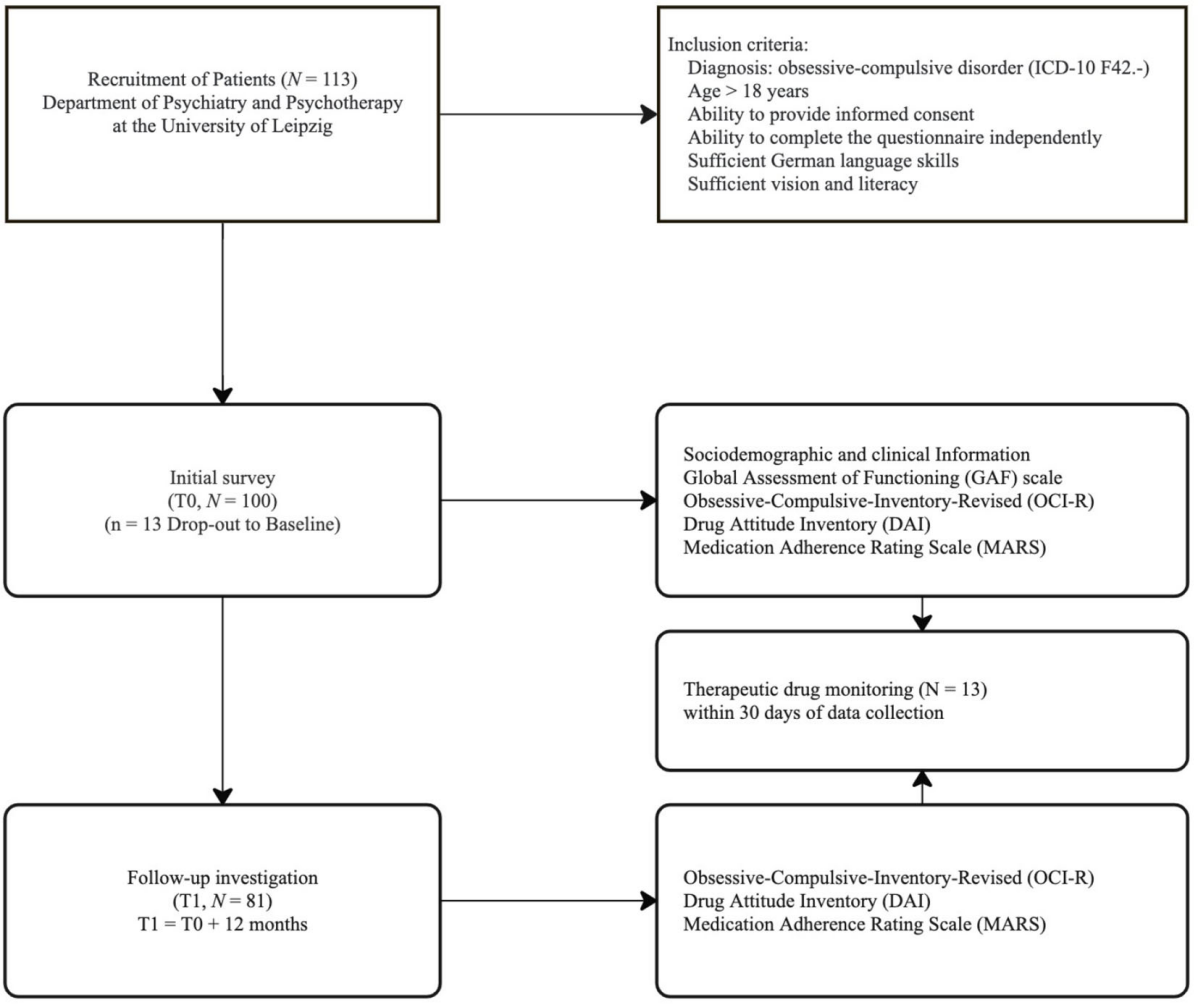
Study flow chart.

The initial investigation included all evaluation tools mentioned below. A total of *N* = 100 individuals completed this assessment in the initial survey T0 and were enrolled in the study. A total of *N* = 81 (81%) individuals took part in the follow-up investigation T1. **Figure 1** provides the study flow chart. A subsample of participants (N = 13) who provided consent for blood sampling and had appointments scheduled within the study timeframe were included in the TDM subgroup. This pragmatic selection was based on feasibility considerations and ethics approval constraints.

#### Sociodemographic and clinical information

First, sociodemographic information (e.g., participants’ ages, genders, marital statuses, educational levels, employment statuses, and parental statuses) was collected. Additionally, clinical data (e.g., the amount, usage, and duration of the prescribed psychopharmacotherapy) were gathered.

#### Functional level and severity of symptoms

The functional level was assessed using the Global Assessment of Functioning (GAF) scale (17). Axis V of the Diagnostic and Statistical Manual of Mental Disorders, Fifth Edition, evaluates a patient’s psychosocial functioning in terms of psychological, social, and occupational performance (18). The scale ranges from 1 to 100, with higher scores indicating better functioning levels. In this study, the patients’ treating psychiatrists recorded the scores. The current severity of obsessive-compulsive symptoms was measured using the Obsessive-Compulsive-Inventory-Revised, German Adaptation (OCI-R) (19, 20). This self-report questionnaire consists of 18 items rated on a 5-point Likert scale and provides scores across six subscales (e.g., washing, checking, ordering, obsessing, hoarding, and neutralizing), with a total score ranging from 0 to 72. Higher scores indicate greater symptom severity.

#### Drug attitude and medication adherence behavior

The Drug Attitude Inventory (DAI) (21) was used to assess attitudes toward medications. This self-report scale consists of ten items in a dichotomous response format and represents a brief versionof the DAI-30. Six items reflect positive attitudes toward pharmacotherapy, while the remaining items reflect negative attitudes. The total score ranges from –10 to 10, with scores >0 indicating a positive attitude.

The Medication Adherence Rating Scale (MARS) (22) was also used to evaluate adherence. This 10-item yes/no self-report instrument was developed based on the Drug Attitudes Inventory and the Morisky Medication Adherence Scale (MMAS-4) (23). MARS is divided into three parts: ‘medication adherence behavior’ (Items 1–4, equivalent to the Morisky Medication Adherence Scale), ‘attitude toward taking medication’ (Items 5–8), and ‘negative side effects and attitudes to psychotropic medication’ (Items 9 and 10). The total score ranges from 0 to 10 and reflects the adherence continuum: scores <5 indicate nonadherence, 5–7 partial adherence, and ≥8 good adherence (24). The questionnaire was adapted to the sample studied, specifically considering the diagnostic inclusion criterion (i.e., OCD).

Moreover, TDM, specifically blood sample analysis, was used in a subset (*N* = 13) to directly assess adherence to the prescribed psychopharmacotherapeutic regimen. Results obtained within 30 days of data collection were included. The selected therapeutic reference range corresponds to the values suggested by the Consensus Guidelines for Therapeutic Drug Monitoring in Neuropsychopharmacology: Update (25).

#### Helpful strategies to assure medical adherence

The study encompassed various behavioral modification strategies (based on clinical experience and research conducted in the specialized OCD outpatient clinic of the department) and their application for supporting adherence: intake of medication before and after specific activities, intake of medication at the same time every day, setting alarms as reminders, receiving text messages as reminder, and recording medication intake on video and sharing it with someone else. Participants could also propose additional strategies. These strategies were evaluated using a 5-point Likert scale from 1 = “strongly disagree” to 5 = “strongly agree”. Higher scores indicated greater participant agreement.

### Statistical analysis

Descriptive statistics were used to summarize sociodemographic and clinical characteristics. A paired *t*-test was conducted under the assumption of normality, given the sample size (*N* > 30), to compare adherence over time (initial survey T0 and follow-up investigation T1) (26). In addition, potential differences in adherence were examined between participants receiving standard antidepressant therapy and those undergoing pharmacological augmentation. Since both groups violated the assumptions of normality, as indicated by the Shapiro–Wilk test (*p* <.05), and the subsample size was relatively small (*N* < 30), a nonparametric Mann–Whitney U test was conducted. The distributions did not differ significantly between the two groups (Kolmogorov–Smirnov test, *p* >.05).

To explore group differences in medication attitudes (DAI total scores) across sociodemographic characteristics (e.g., education, employment status, gender, and relationship status), Kruskal–Wallis tests were applied, as the data were not normally distributed across comparison groups. Due to the very small number of participants in the “diverse” gender and “widowed” relationship status categories (n = 1), these subgroups were excluded from the comparative analyses.

The DAI and MARS scores were the dependent variables. Multiple linear regression was conducted in two blocks to identify predictors of medication adherence after verifying the assumptions (e.g., independence and normal distribution of errors, homoscedasticity, and the absence of multicollinearity). In the first step, sociodemographic data (e.g., age, gender, and educational level) were entered as predictors, while clinical features (e.g., duration from symptom onset to diagnosis, GAF score, and OCI-R score) were predictors in the second step. The dependent variable was medication adherence, measured through indirect methods (i.e., MARS). A two-tailed *α* <.05 was applied for all statistical tests. Statistical analyses were performed using the Statistical Package for Social Sciences (IBM SPSS Statistics 29.0 for Windows; SPSS Inc, Chicago, IL). Data are available upon request.

## Results

### Sample characteristics

The sample consisted of *N* = 100 patients during the initial survey T0, of whom *N* = 81 (81%) participated during the follow-up investigation T1. Demographic characteristics are presented in **Table 1**. Most (57.0%, *n* = 57) of the participants were female, 42% were male (*n* = 42), and 1.0% (*n* = 1) were diverse with an age range of 18–82 years (*M* = 40.04, *SD* = 13.74).

**Table 1:** Participants’ sociodemographic characteristics (N = 100).

Variable	Participants T0
Gender, *n* (%)	
Female	57 (57.0)
Male	42 (42.0)
Divers	1 (1.0)
Age	
18–25	14 (14.0)
26–30	15 (15.0)
31–40	30 (30.0)
41–50	18 (18.0)
51–60	17 (17.0)
>60	6 (6.0)
Relationship status, *n* (%)	
Single	38 (38.0)
Married or living with a partner	54 (54.0)
Divorced or separated	7 (7.0)
Widowed	1 (1.0)
Educational level, *n* (%)	
Secondary education	15 (15.0)
Postsecondary non-tertiary education	15 (15.0)
Vocational education	34 (34.0)
Bachelor’s, master’s, doctoral, or equivalent level	36 (36.0)
Occupation, *n* (%)	
Apprentice/student	15 (15.0)
Employee/official	45 (45.0)
Incapacitated for work	9 (9.0)
Unemployed	8 (8.0)
Retiree	23 (23.0)

### Functional level and severity of symptoms

The severity of illness was assessed using the GAF scale, with amean GAF score of 68.69 (*SD* = 12.51). The mean total score for the Obsessive-Compulsive-Inventory-Revised was 24.53 (*SD*=14.16). The subscale mean score were as follows: washing (*M* = 3.85; *SD* = 3.98), checking (*M* = 5.31; *SD* = 3.65), ordering (*M*= 3.94; *SD* = 3.70), obsessing (*M* = 6.18; *SD* = 3.57), hoarding (*M*= 2.65; *SD* = 2.78), and neutralizing (*M* = 2.60; *SD* = 3.18).

### Psychopharmacotherapy and medication adherence behavior

#### Initial survey T0

Among the total sample of *N* = 100, most participants reported taking psychiatric medication (*n* = 86, 86.0%). The majority (*n* = 81, 94.2%) were treated with SSRIs, most frequently sertraline (*n* = 46, 53.5%), followed by escitalopram (*n* = 13, 15.1%), citalopram (*n* = 10, 11.6%), paroxetine (*n* = 6, 7.0%), fluoxetine (*n* = 5, 5.8%), and fluvoxamine (*n* = 1, 1.2%). No participant received a combined SSRI–clomipramine regimen. Regarding augmentation strategies, antipsychotic augmentation was reported in 12 cases (13.9%), including quetiapine (*n* = 8, 9.3%), risperidone (*n* = 2, 2.3%), and aripiprazole (*n* = 2, 2.3%). Four participants (4.7%) received additional psychopharmacological agents (e.g., lithium, mirtazapine, amineurin). Most patients (*n* = 70, 83.7%) received monotherapy, while 15 (17.4%) received polypharmacy with two or more psychotropic drugs. Almost one-third of respondents (30%) reported taking their medication for up to five years, 26% for up to ten years, and 19% for more than ten years. One-quarter (25%) reported using medication for up to one year. Fourteen patients (16.3%) underwent medication adjustments during treatment.

Concerning the reported attitudes toward medication, the mean DAI score for all participants (*N* = 82) was 2.05 (*SD* = 4.30), with a range from –10 to +10. However, three items showed negative mean values, which indicated a lack of agreement among the respondents: Item 9 (‘My thoughts are clearer on medication’, *M* = –0.13, *SD* = 1.00), Item 10 (‘By staying on medication, I can prevent getting sick’, *M* = –0.44, *SD* = 0.90), and Item 6 (‘I take my medication only when I am sick’, *M* = –0.13, *SD* = 1.00). Notably, Item 6 was reverse-coded, so a negative score reflected agreement with the statement. Across the MARS questionnaire, the mean score was 6.33 (*SD* = 1.72), with a range of 2–10 (*N* = 82) and a median of 6. For further details on the descriptive distribution, see **Table 2**.

**Table 2:** Descriptive statistics for MARS and DAI.

Scale / Subscale	*n*	*M*	*SD*	*Var*	*Min/Max*
MARS	82	6.33	1.715	2.940	2/10
Medication adherence behavior	85	3.22	.943	.890	0/4
Attitude toward taking medication	84	1.92	1.111	1.234	0/4
Negative side effects and attitudes to psychotropic medication	85	1.19	.715	.512	0/2
DAI	82	2.05	4.297	18.467	−10/+10

*M*, mean; *SD*, standard deviation; *Var*, variance; *Min*, minimum; *Max*, maximum.

To further characterize individuals more or less likely to adhere to pharmacological treatment, we conducted a series of Kruskal–Wallis tests to explore whether medication attitudes (DAI total score) differed significantly across key sociodemographic groups. No statistically significant differences were observed with regard to educational level (*H*(4) = 2.60, *p* = .628), employment status (*H*(5) = 7.82, *p* = .167), marital status (*H*(3) = 6.37, *p* = .095), or gender (*H*(2) = 1.33, *p* = .513). However, several descriptive trends emerged: individuals with higher educational attainment and those attending university tended to report more favorable medication attitudes, whereas lower scores were observed among participants in vocational training. Similarly, those in stable relationships showed more positive attitudes compared to single or divorced participants. Gender, in contrast, was not a distinguishing factor, with nearly identical mean ranks for men and women. These findings suggest that while no strong sociodemographic predictors of medication attitudes were detected in this sample, certain subgroup patterns may warrant further investigation in larger studies.

Participants who received antipsychotic augmentation differed significantly from the rest of the sample in both overall DAI scores (*U* = 255.00, *Z* = -2.189, *p* <.05) and MARS scores (*U* = 240.00, *Z* = -2.403, *p* <.05). Regarding the MARS subscales, a significant group difference was observed for Attitude toward taking medication (*U* = 193.50, *Z* = -3.166, *p* <.01), whereas no significant differences were found for the subscales Medication adherence behavior and Negative side effects.

#### Follow-up investigation T1

In total, *N* = 81 individuals participated in the second survey, with most (*n* = 70, 86.4%) reporting psychiatric medication use. Consistent with the first survey, most (*n* = 66, 94.3%) received SSRI treatment. Sertraline remained the most prescribed SSRI (*n* = 40, 57.1%), followed by escitalopram (*n* = 12, 17.1%), citalopram (*n* = 7, 10.0%), paroxetine (*n* = 3, 4.3%), fluoxetine (*n* = 3, 4.3%), and fluvoxamine (*n* = 1, 1.4%). Only two respondents (2.9%) reported undergoing medication adjustments. In five cases (7.14%), SSRI therapy was augmented with either quetiapine (*n* = 4, 5.7%) or aripiprazole (*n* = 1, 1.4%). No SSRI–clomipramine combinations were reported. No significant difference between the initial survey T0 and the follow-up investigation T1 was found in the attitudes toward medication (i.e., DAI: *t*[66] = 1.876, *p* = .065) or in adherence behavior (i.e., MARS: *t*[64] = .251, *p* = .803).

### Therapeutic drug monitoring

TDM (i.e., blood samples) was used to measure medication adherence directly. In total, *n* = 11 (84.6%) individuals showed plasma drug concentrations within the therapeutic reference range for the prescribed medication, whereas *n* = 2 (15.4%) displayed subtherapeutic drug concentrations. Both individuals reported partial adherence on the Medication Adherence Rating Scale (MARS; scores of 7 and 6) and expressed moderately positive attitudes toward pharmacotherapy on the Drug Attitude Inventory (DAI; both scores = 4). These self-reported results are consistent with their subtherapeutic serum profiles, suggesting a coherent pattern across subjective and objective adherence measures.

### Predictors of medication adherence

#### Block 1: Sociodemographic Data (i.e., age, gender, and educational level)

The results indicated that the predictors did not significantly explain the model (*F*[3, 74] = 1.096, *p* = 0.356), as they accounted for only 4.3% of the variance (*R* ^2^ = 0.043). None of the predictors were significantly associated with medication adherence.

#### Block 2: Clinical features (i.e., duration from symptom onset to diagnosis, GAF score, and OCI-R score)

In the second block, clinical characteristics were added to the model. This block did not significantly improve the predictive power (Δ*F*[3, 61] = 0.558, *p* = 0.762), as it only slightly increased the explained variance to 4.5% (*R* ^2^ = 0.045), indicating a low goodness of fit (27). Similarly, none of the predictors were significantly associated with medication adherence (see **Table 3**).

**Table 3:** Prediction of medication adherence by age, gender, and educational level (Step 1) and duration from symptom onset to diagnosis, GAF score, and OCI-R score (Step 2).

Predictor	*B*	*SE*	*ß*	*p*
Step 1				
Gender	.268	.363	.084	.464
Age	.022	.014	.181	.125
Educational level	.057	.190	.038	.832
Step 2				
Duration from symptom onset to diagnosis	.006	.019	.037	.758
GAF score	–.005	.020	–.036	.818
OCI-R score	.002	.016	.014	.918

*N* = 78. *B* = unstandardized coefficient; *SE* = standard error; *ß* = standardized coefficient; *p* = p-value. *R* ^2^ = .043 for Step 1 (*p* = .356); Δ *R* ^2^ = .002 for Step 2 (*p* = .762).

### Helpful strategies for ensuring drug adherence

Regarding behavioral modification strategies to ensure medication adherence, two suggestions received strong endorsement and frequent application by the respondents: ‘intake of medication before and after specific activities’ and ‘intake of drugs at the same time every day’. The mean score for coupling drug intake with specific activities received the highest level of agreement, with *n* = 33 (38.0%) of the sample strongly supporting this strategy. Notably, the suggestion ‘film one’s own medication intake and send it to someone’ received the lowest approval, with *n* = 74 (86.0%) strongly disagreeing and only one strongly agreeing (*n* = 1, 1.2%). These observations were reflected in the implementation of these strategies in everyday life, with most respondents (*n* = 56, 65.1%) stating that their medication intake was associated with daily activities. Additionally, *n* = 28 (32.6%) reported taking their medication at the same time every day. Twenty respondents provided free-text responses. Of these, *n* = 7 (35%) mentioned the benefit of placing their medication alongside commonly used objects (e.g., glasses and a bedside table). Six participants (30%) linked their medication intake to meals or bedtime routines. Other strategies mentioned included taking psychopharmaceutical drugs together with other medications or using pill dispensers.

## Discussion

The results of this study provide a unique insight into medication adherence in people with OCD, a frequently underrepresented population in adherence research.

In the present study, the participants reported mild impairments in psychological, social, and occupational functioning despite the presence of clinically significant OCD symptoms (18, 28). Obsessive thoughts and checking were the most common while washing, hoarding and neutralizing, whereas washing, hoarding, and neutralizing were less frequently reported. The combination of moderate GAF scores and clinically relevant OCI-R scores indicated that, although individuals functioned relatively well in daily life, OCD symptoms continued to cause substantial distress and interfered with everyday activities.

In light of these findings, understanding adherence to prescribed pharmacological treatments becomes essential. Most participants (over 80%) were prescribed SSRIs, and a smaller subset (17.4%) received antipsychotic augmentation. Previous research has shown that antipsychotic augmentation (e.g., quetiapine, risperidone, or aripiprazole) may improve treatment outcomes in OCD that is refractory to treatment (29–32). Current treatment guidelines distinguish between combination strategies and augmentation. While antipsychotic augmentation (e.g., with aripiprazole or risperidone) is formally recommended after insufficient response to SSRI or clomipramine monotherapy, combining an SSRI with clomipramine is typically considered after multiple failed monotherapy trials and is supported by lower evidence strength, while also posing the risk of clinically severe drug interactions (14).

One-fourth (24.4%) of the participants reported good medication adherence, 62.2% reported partial adherence, and 13.4% reported nonadherence based on self-reported questionnaire data. No significant change was observed in adherence levels over a one-year period, which indicated a stable pattern of adherence behavior within that timeframe. Consequently, approximately three-quarters of the participants did not take their medication as prescribed (i.e., partial or nonadherence). This finding is consistent with previous research, which has shown that 50% or more of patients in long-term psychiatric treatment either fail to fully adhere to their regimen or discontinue medication entirely (33–35). Interestingly, participants who were undergoing pharmacological augmentation differed significantly from the rest of the sample in both overall medication attitudes (DAI) and self-reported adherence (MARS). Notably, this difference was primarily driven by the subscale Attitude toward taking medication, suggesting greater ambivalence among those receiving augmentation. While actual adherence behavior did not differ significantly, the more negative attitudes in this group may reflect perceptions of treatment chronicity, reduced outcome expectations, or internalized stigma – particularly given the association of antipsychotics with more severe psychiatric conditions. Previous research has shown that internalized stigma is a critical factor that negatively impacts medication adherence, particularly in patients with OCD (36, 37). Higher levels of self-stigma are significantly correlated with lower adherence, likely because stigma and misconceptions about psychiatric disorders act as barriers to seeking and completing treatment. Taken together, these findings underscore the importance of addressing both emotional and cognitive responses to complex treatment regimens, as well as implementing stigma-reduction strategies to improve adherence and optimize long-term outcomes – especially in patients with treatment-resistant OCD.

Interestingly, 84.6% of the participants for whom TDM was available had drug levels within the therapeutic range, while only 15.4% had subtherapeutic levels. TDM is an objective measure of adherence, but it may not always correlate perfectly with self-reported medication behavior due to factors such as irregular dosing, metabolic variability, or individual differences in drug absorption and pharmacokinetics (25, 38, 39). In the present study, both individuals with subtherapeutic levels reported partial adherence and only moderately positive medication attitudes. This pattern reflects a clinically plausible profile – where medication is not entirely rejected but instead is taken inconsistently or without full conviction. Such cases highlight the nuanced continuum between behavioral and attitudinal adherence, and underscore the utility of TDM as a complementary tool to detect suboptimal intake that may otherwise remain undetected by self-report alone. The overall high proportion of therapeutic drug levels in this subgroup may also indicate optimized dosing and pharmacological monitoring, which may mitigate side effects and enhance adherence (39, 40).

The study also revealed significant concerns among participants regarding their prescribed medication. Notably, no significant difference was found in DAI-10 scores during the one-year follow-up investigation. Approximately 24.4% were dissatisfied with their current pharmacological treatment, while 72.1% were unsure whether it would prevent relapse and improve cognitive function. While the effects of SSRIs on cognition remain unclear and the results are inconsistent at present (41–43), SSRIs have been shown to reduce symptoms and prevent relapse (44). Long-term use of SSRIs can stabilize symptoms, reduce the likelihood of relapse, and improve quality of life (45–47). However, many participants (55.8%) strongly believed that medication is only necessary during acute illness episodes. Particularly concerning are previous findings showing an association between lower positive drug attitudes and poor adherence (48). Poor adherence alone can lead to symptom relapse, increased hospitalizations, and elevated suicide risk (49–51). Therefore, addressing these attitudinal and belief-based barriers is crucial for improving medication adherence and preventing long-term negative outcomes.

Regarding adherence predictors, no significant relationship was found between sociodemographic factors (e.g., age, gender, and education) or clinical factors (e.g., duration from symptom onset to diagnosis, GAF score, and OCI-R score). This finding is consistent with the existing literature, which suggests that sociodemographic factors are generally inconsistent predictors of adherence in psychiatric populations (33, 52). In contrast, certain clinical factors, such as symptom severity and illness duration, have demonstrated predictive value in other studies (53–55), highlighting the need for a more nuanced approach that considers a broader range of psychosocial variables (e.g., insight into illness, social support, and treatment beliefs or credibility).

Strategies to improve adherence primarily involve behavioral modification. The participants supported strategies such as coupling medication intake with daily activities (e.g., meals and bedtime routines). These approaches highlight the role of habit formation in reducing the cognitive load associated with remembering to take medication (56, 57, 58). In addition, the consistent timing of medication intake, particularly for psychopharmaceuticals, may enhance treatment effectiveness by reinforcing routines (59–61). However, external monitoring strategies (e.g., filming medication intake) have been largely rejected. Recent research suggests that while digital tools can be beneficial, overly invasive methods may diminish patients’ sense of autonomy and create discomfort (62–64).

### Strengths and limitations

This study makes a significant contribution to advancing the understanding of medication adherence in OCD, an underexplored group in adherence research. The large sample size relative to the population studied, combined with follow-up assessments, strengthens the robustness of the findings. Furthermore, using direct and indirect methods for measuring medication adherence enhances the validity and reliability of the results. Nevertheless, several limitations to this study should be considered when interpreting the results. First, this study could not establish causation, so all results must be regarded as associations. Second, with medication adherence, operationalization is only possible to a limited extent, especially when self-report questionnaires are used, as in the present study. Third, there is a substantial need to investigate how existing psychotherapeutic treatment may influence medication adherence. Fourth, TDM was only available in a subgroup of the sample. Fifth, insight – a well-established predictor of medication adherence in psychiatric populations – was not formally assessed in this study (49). This represents a notable limitation, as insight can substantially influence a patient’s willingness to initiate, continue, and adhere to pharmacological treatment. While most individuals with OCD retain at least partial awareness of the irrational nature of their obsessions (18), the degree of insight can vary widely and may fluctuate over the course of the illness. In the present study, only self-report instruments were administered, and none specifically measured insight. Without a standardized psychometric assessment, it was not possible to explore potential associations between insight and adherence in our sample. Future research should incorporate validated measures of insight to clarify its potential role as a moderator or mediator of adherence and to inform the development of tailored adherence-enhancing interventions.

## Conclusions

This study investigated medication adherence in individuals with OCD, a group often underrepresented in research. The findings revealed that partial adherence was relatively common. The participants experienced significant OCD symptoms and moderate impairments in various life domains despite receiving appropriate treatments such as SSRIs and, in some cases, augmentation with antipsychotics.

The study underscores the potential of TDM not only as a tool for optimizing pharmacotherapy and improving adherence in complex cases, but also for objectively detecting suboptimal medication intake that may not be captured by self-report. Nevertheless, TDM results may not always perfectly align with actual medication behavior due to various influencing factors. Participants’ notable concerns about medication side effects and doubts regarding efficacy highlight the importance of directly addressing these issues to improve adherence.

Behavioral strategies (e.g., integrating medication intake into daily routines) are promising for enhancing adherence by leveraging habitual behavior and aligning medication schedules with circadian rhythms. However, external monitoring methods, perceived as intrusive, are less favorably received.

This study underscores the importance of personalized, adaptive approaches to treatment and adherence in OCD, emphasizing the role of TDM, patient education, and the establishment of supportive routines that both empower patients and address their concerns about medication.

## Data Availability

The raw data supporting the conclusions of this article will be made available by the authors, without undue reservation.
